# A Mild Impairment of Mitochondrial Electron Transport Has Sex-Specific Effects on Lifespan and Aging in Mice

**DOI:** 10.1371/journal.pone.0026116

**Published:** 2011-10-10

**Authors:** Bryan G. Hughes, Siegfried Hekimi

**Affiliations:** Department of Biology, McGill University, Montréal, Canada; Brown University, United States of America

## Abstract

Impairments of various aspects of mitochondrial function have been associated with increased lifespan in various model organisms ranging from *Caenorhabditis elegans* to mice. For example, disruption of the function of the ‘Rieske’ iron-sulfur protein (RISP) of complex III of the mitochondrial electron transport chain can result in increased lifespan in the nematode worm *C. elegans*. However, the mechanisms by which impaired mitochondrial function affects aging remain under investigation, including whether or not they require decreased electron transport. We have generated knock-in mice with a loss-of-function *Risp* mutation that is homozygous lethal. However, heterozygotes (*Risp^+/P224S^*) were viable and had decreased levels of RISP protein and complex III enzymatic activity. This decrease was sufficient to impair mitochondrial respiration and to decrease overall metabolic rate in males, but not females. These defects did not appear to exert an overtly deleterious effect on the health of the mutants, since young *Risp^+/P224S^* mice are outwardly normal, with unaffected performance and fertility. Furthermore, biomarkers of oxidative stress were unaffected in both young and aged animals. Despite this, the average lifespan of male *Risp^+/P224S^* mice was shortened and aged *Risp^+/P224S^* males showed signs of more rapidly deteriorating health. In spite of these differences, analysis of Gompertz mortality parameters showed that *Risp* heterozygosity decreased the rate of increase of mortality with age and increased the intrinsic vulnerability to death in both sexes. However, the intrinsic vulnerability was increased more dramatically in males, which resulted in their shortened lifespan. For females, the slower acceleration of age-dependent mortality results in significantly increased survival of *Risp^+/P224S^* mice in the second half of lifespan. These results demonstrate that even relatively small perturbations of the mitochondrial electron transport chain can have significant physiological effects in mammals, and that the severity of those effects can be sex-dependent.

## Introduction

Mitochondrial oxidative phosphorylation is essential to aerobic organisms, as it provides the bulk of usable energy in the form of ATP. In humans, mitochondrial defects are often studied in the context of severe deficits in function, resulting in serious negative health consequences. These mitochondrial diseases can be caused by mutations to a wide variety of mitochondrial and nuclear-encoded genes [1], [2]. Impairments of mitochondrial function have also been found to accompany numerous age-dependent diseases, such as atherosclerosis, type 2 diabetes and various neurodegenerative disorders [3]–[5], as well as aging in general, even in the absence of overt disease. This has led to a widespread belief that mitochondrial dysfunction plays a causative role in aging [6]. However, a variety of interventions that impair mitochondrial function in model organisms have been shown to be capable of increasing lifespan. In fact, one of the very first aging genes to be identified in the nematode worm *Caenorhabditis elegans* was *clk-1*, which encodes a conserved mitochondrial enzyme required for the biosynthesis of ubiquinone, a crucial electron carrier of the mitochondrial electron transport chain (ETC) [7], [8]. Since then, several mutations that affect mitochondrial proteins have been found to increase lifespan, including mutations in subunits of the complexes of the mitochondrial respiratory chain [9]–[11]. In addition, RNA interference (RNAi) against various components of mitochondria, including respiratory chain subunits, have also been shown to extend lifespan [11]–[14], albeit by a mechanism that appears to be different from that triggered by the mutations [11].

In the past few years, longevity-promoting effects of mitochondrial dysfunction have also been found in other species. RNAi knockdown of components of the mitochondrial electron transport chain has been shown to extend lifespan in *Drosophila* [15], and two genetic interventions causing limited mitochondrial dysfunction have been found to increase lifespan in mice: (1) the knockout of *Surf1*, encoding a cytochrome *c* oxidase assembly factor [16] and (2) heterozygosity for *Mclk1* (*Mclk1* ^+/−^), the mammalian homologue of the *C. elegans* aging gene *clk-1* [17], [18]. It therefore appears that the ability of mitochondrial impairment to extend lifespan is conserved across species.

Although the effect of mitochondrial dysfunction on longevity tentatively appear to be conserved, the underlying mechanisms remain unclear. For example, in mice, both *Surf1* knockouts and *Mclk1* heterozygotes have impaired ETC activity, as expected from the known functions of the genes [16], [19]. However, knocking out *Surf1* did not affect mitochondrial membrane potential, leading investigators to suggest that alterations to mitochondrial bioenergetics may not lie behind the increased lifespan. Their finding of increased longevity is also complicated by the facts that (1) another line of *Surf1* knockout mice is dramatically short-lived [20] and (2) *Surf1* mutations have severe negative health effects in humans [21]. In contrast to *Surf1* knockout mice, long-lived *Mclk1* ^+/−^ mice exhibit clear mitochondrial dysfunction characterized by impairments to both ETC activity and mitochondrial respiratory rate. However, it is difficult to single out the ETC defect as the cause for the observed extension of lifespan in these mice because of (1) the importance of ubiquinone for other systems beyond the ETC [22] and (2) *Mclk1^+/−^* mice show widespread changes in levels of oxidative stress, which has the potential to effect lifespan [19], [23].

The ‘Rieske’ iron-sulfur protein (RISP, also known as the ubiquinol-cytochrome c reductase rieske iron-sulfur polypeptide 1, UQCRFS1) is one of the core components of the ubiquinol-cytochrome *c* reductase (complex III) of the mitochondrial electron transport chain. RISP contains a 2Fe-2S prosthetic group that accepts a single electron from ubiquinol and then transfers it to the cytochrome *c*_1_ subunit, from which it is transferred to cytochrome *c* [24], [25]. The protons obtained from the oxidation of ubiquinol are transferred to the inter-membrane space, thereby contributing to the proton-motive force required for the phosphorylation of ADP to ATP. A mutation in *isp-1*, the *C. elegans* homologue of *Risp*, extends lifespan [9]. The life-extending *isp-1* mutation is a single base substitution that changes a conserved proline into a serine. The affected proline is close to the residues that hold the 2Fe-2S group in place, and may therefore play an important structural role [26]. There is some evidence that the mutant protein retains partial function: residual NADH-cytochrome *c* reductase enzymatic activity and cyanide-sensitive oxygen consumption of whole worms suggests that the ETC is not completely inhibited [9], [11].

In order to better understand how mitochondrial function can modulate lifespan in mammals we have generated a “knock-in” mouse strain that carries a proline-to-serine point mutation (P224S) at the same conserved proline as in the *C. elegans* long-lived *isp-1* mutant. Manipulating *Risp* should affect ETC activity very directly in contrast to *Surf1^−/−^* and *Mclk1^+/−^* mice. We found that the *Risp^P224S^* allele is homozygous-lethal in mice but that heterozygous mice (*Risp^+/P224S^*) had lower RISP protein levels as well as partially impaired complex III activity and lower mitochondrial oxygen consumption. When young, these mice exhibited no overt health deficits. We also observed no effects on biomarkers of oxidative stress at any age. Yet, *Risp^+/P224S^* males had a decreased average lifespan relative to wild-type littermates. In contrast, the average lifespan of *Risp^+/P224S^* females was unaffected. However, those mutant females that survived up to the wild-type median lifespan subsequently lived significantly longer on average than the remaining wild type females. In spite of these lifespan differences between the sexes, analysis of Gompertz parameters showed that *Risp* heterozygosity decreased the rate of increase of mortality with age and increased the intrinsic vulnerability to death in both sexes. However, the intrinsic vulnerability was increased more dramatically in males, which resulted in their shortened lifespan.

## Results

### Homozygous *Risp^P224S/P224S^* mice are not viable but heterozygous *Risp^+/P224S^* mice are healthy and fertile

We used the techniques of homologous recombination in embryonic stem cells and Flp/FRT site-specific recombination to generate a line of mice carrying a *Risp* mutation identical to that found to increase lifespan in *C. elegans* (see Materials and Methods). The mice carried a *Risp* allele with the same proline-to-serine change at a conserved proline that is mutated in *isp-1(qm150)* mutant worms. The only other difference between the knock-in allele and the wild-type is the single FRT site remaining downstream of the *Risp* gene. We hoped that residual functionality of the mutant protein, similar to what was found in *C. elegans*, would allow for the production of viable homozygous mice. However, none out of almost 200 offspring of *Risp* ^+/*P224S*^×*Risp* ^+/*P224S*^ matings were found to be homozygous for the knock-in allele, indicating that the mutation is homozygous-lethal in mice. Supporting this, the ratio of wild-type versus heterozygotes fits the Mendelian ratio expected if the one-quarter of offspring that are homozygous are not viable (38∶62 percent, p>0.05 vs. an expected ratio of 33∶66 by the chi-square test). We did not investigate the lethality phenotype further.

**Figure 1:**
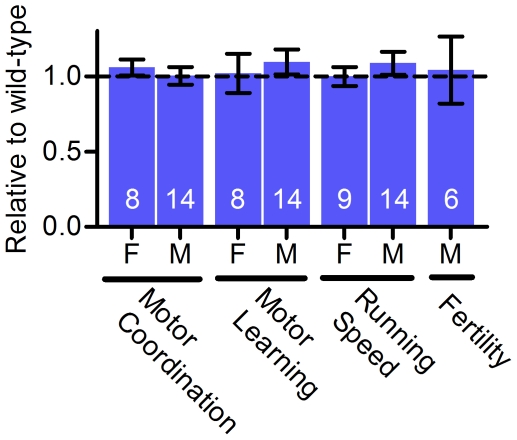
Young (3 months old) *Risp^+/P224S^* mice have unimpaired physiological performance. The sex of the mice (F for female, M for male), as well as the type of test, are indicated below each bar. Mice were tested for motor coordination (average of 3 trials after 2 days of training on a Rota-Rod), motor learning (Rota-Rod performance on day 3 relative to day 1), running speed (the maximum speed mice could maintain on a powered treadmill) and male fertility (number of pups sired by *Risp* ^+/P224S^ males with wild-type females). All measurements were made in wild-type/heterozygote littermate pairs, and results are expressed as the effect of *Risp* heterozygosity relative to wild-type within each pair. Sample sizes (number of littermate pairs) are shown superimposed on each bar. Actual (not normalized) values are shown in Table S2.

Subsequent breeding was carried out with *Risp* ^+/*P224S*^ males being mated to *Risp^+/+^* females. These crosses resulted in the expected Mendelian ratio (50∶50 for wild-type∶heterozygotes, p>0.05 by the chi-square test), suggesting that *Risp* ^+/*P224S*^ mice do not suffer from significant embryonic lethality. Several tests of physical and neurological performance (motor coordination, motor learning, running speed and male fertility) revealed no effect of *Risp* heterozygosity on young mice (Figure 1), and body and organ weights were the same as wild-type (Table S1). To obtain a rough measure of female fertility, we compared the breeding records for crosses of heterozygous males to wild-type females with those for crosses of heterozygotes with heterozygotes (correcting for the absence of homozygous mutant mice). By this measure, no deleterious effect of *Risp* heterozygosity on female fertility was observed (wild-type female breeders produced 22±4.4 offspring vs. 24.9±4.2 from heterozygous breeders).

### Decreased RISP protein and complex III enzymatic activity in *Risp^+/P224S^* mice

**Figure 2:**
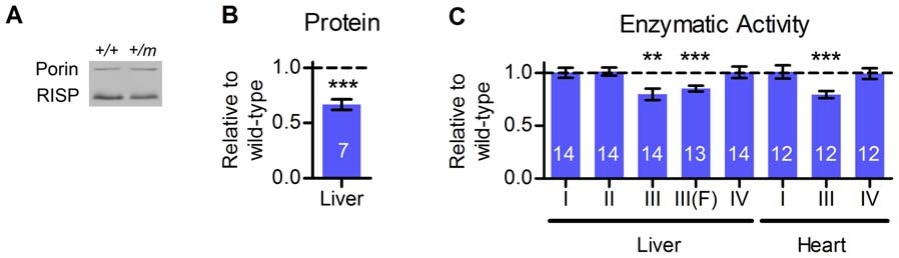
Young *Risp* ^+/P224S^ mice have decreased RISP protein and complex III enzymatic activity. (**A**) A representative western blot showing RISP protein levels in mitochondria isolated from liver of 3-month-old female adults. A wild-type/heterozygous littermate pair is shown (‘*m*’ denotes the knock-in mutant allele P224S). (**B**) Densitometry was used to quantify RISP relative to porin. All measurements were made in wild-type/heterozygote littermate pairs, and results are expressed as the effect of *Risp* heterozygosity relative to wild-type within each pair. (**C**) Enzymatic activity of complexes of the mitochondrial ETC (complex I, II, III or IV) was determined in disrupted mitochondria, using appropriate substrates and inhibitors to isolate the activity of each complex. All measurements were made in wild-type/heterozygote littermate pairs, and results are expressed as the effect of *Risp* heterozygosity relative to wild-type within each pair. All results are for male mice, except where indicated (as F, for female). Actual densitometry values for (A) and enzymatic activities for all samples (C) are shown in Table S2. Sample sizes (i.e., number of wild-type/heterozygous littermate pairs) are shown superimposed on each bar. ‘**’ denotes p = 0.001 to 0.01 and ‘***’ denotes p<0.001 vs. 1.0.

Mitochondria isolated from the liver of young adult *Risp^+/P224S^* mice were found by western blotting to contain less RISP protein than wild-type controls (Figure 2A). Although quantification by western blotting is known to have its limitations, we estimated that the level of RISP in *Risp* ^+/P224S^ mutants was reduced to two-thirds of the wild-type level (Figure 2B). These observations suggest that the mutant RISP is unstable, or that complexes that incorporate mutant RISP are turned over more rapidly. This is consistent with the finding that *Risp* point mutations in this polypeptide stretch decrease the steady state level of RISP protein in the yeast *Saccharomyces cerevisiae* [26].

The enzymatic activity of complex III in the liver of adult *Risp^+/P224S^* mutants was decreased by 20 and 15 percent in males and females, respectively (Figure 2C). The activity was decreased by 21 percent in the hearts of males (Figure 2C) (female hearts were not examined). Thus the levels of decrease in RISP protein and complex III enzymatic activity in *Risp^+/P224S^* mice appear to be of a comparable magnitude. This suggests that RISP is limiting for complex III activity, and that the decrease in complex III activity is likely not due to incorporation of mutant protein in the complex.

To determine if the impact of the mitochondrial deficit of RISP in *Risp^+/P224S^* mice was specific to complex III as expected, we measured the enzymatic activities of complexes I, II and IV in mitochondria extracted from liver and that of complex I and IV in mitochondria extracted from heart. The activities of these complexes were not affected by *Risp* heterozygosity, neither up- nor down-regulated (Figure 2C). Thus, the decrease in complex III enzymatic activity is likely to be the sole cause of the phenotypes we describe below.

### A substrate-dependent decrease in liver mitochondrial respiration in *Risp^+/P224S^* mice

**Figure 3:**
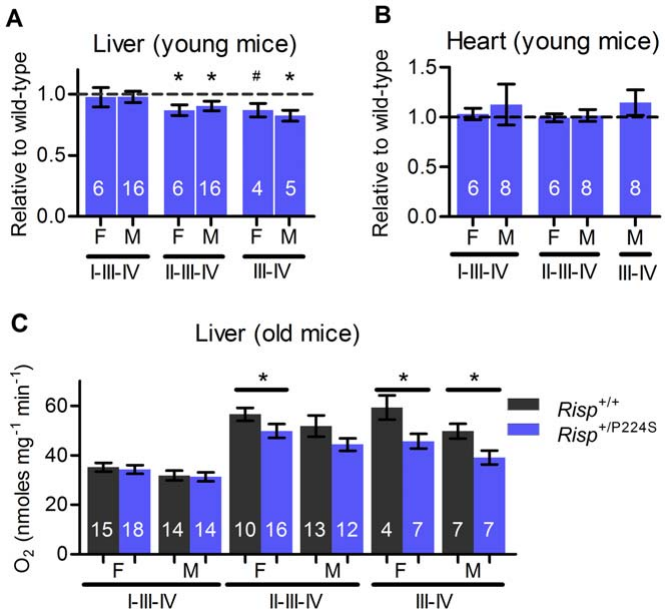
Oxygen consumption of isolated mitochondria in *Risp* ^+/P224S^ mice. Electron paths due to the combination of substrate and inhibitors used are indicated below each bar, as well as the sex (F for female, M for male). (**A**) Mitochondria isolated from liver of 3-month-old mice. All measurements were made in wild-type/heterozygote littermate pairs, and results are expressed as the effect of *Risp* heterozygosity relative to wild-type within each pair. (**B**) Mitochondria isolated from heart of 3-month-old mice. All measurements were made in wild type/heterozygote littermate pairs, and results are expressed as the effect of *Risp* heterozygosity relative to wild-type within each pair. (**C**) Mitochondria isolated from liver of 2-year-old mice. Due to the low probability of both members of wild-type/heterozygote littermate pairs surviving to this age, comparisons were made between the complete group of mice of each genotype. Actual values for (A) and (C) are shown in Table S2. Values for state 4 respiration and respiratory control ratio (state 3/state 4) are shown in Table S3. For all figures, sample sizes are shown superimposed on each bar. ‘*’ denotes p = 0.01 to 0.05 vs. 1.0 or wild-type control, ‘^#^’ p = 0.097 vs. 1.0.

We isolated mitochondria from livers of young and aged mice (3 months and 2 years old, respectively) and measured their rate of oxygen consumption to determine if the deficit of complex III activity described above could affect overall oxidative phosphorylation. Actively respiring mitochondria, in the presence of non-limiting amounts of ADP and substrate, are said to be in state 3. We found that state 3 respiration was not affected when the substrates provided to mitochondria supplied electrons to complex I via NADH (glutamate + malate; in Figure 3 this situation is noted as I-III-IV to indicate the path followed by electrons through the ETC). This was true for mitochondria from the liver of both young and old mice (Figure 3A and 3C; the difference in form between graphs for young and aged mice is because littermate pairs could be analyzed for young, but not old, mice as described in Materials and Methods). In contrast, state 3 respiration supported by succinate, which donates electrons directly to complex II (II-III-IV pathway), was significantly decreased by 10 to 13 percent in mitochondria of *Risp^+/P224S^* mice. The effect did not reach statistical significance in 2-year-old *Risp^+/P224S^* males, but was clearly apparent as a trend, and was similar in magnitude to that observed in the aged *Risp^+/P224S^* females (Figure 3C). We used the ubiquinol-analog duroquinol (DQ) [27] to donate electrons directly to complex III (III–IV pathway), and therefore obtained a state 3 that is independent of both complexes I and II. State 3 respiration was decreased with DQ as substrate in young and old mice (Figure 3A and 3C). Therefore, the complex III impairment described above is sufficient to impair mitochondrial function, but the effect is substrate-dependent. We also measured oxygen consumption of mitochondria from heart. In this tissue, there was no effect of *Risp* heterozygosity, regardless of sex or substrate used (Figure 3B). Average respiratory control ratios were not affected by genotypes, and ranged from 5.0–9.4 for glutamate and malate, 3.4–9.4 for succinate, and 4.4–16.0 for duroquinol, indicating that mitochondria in our preparations were well-coupled and intact (Table S3).

### Metabolic rate is lowered in young male *Risp^+/P224S^* mice

**Figure 4:**
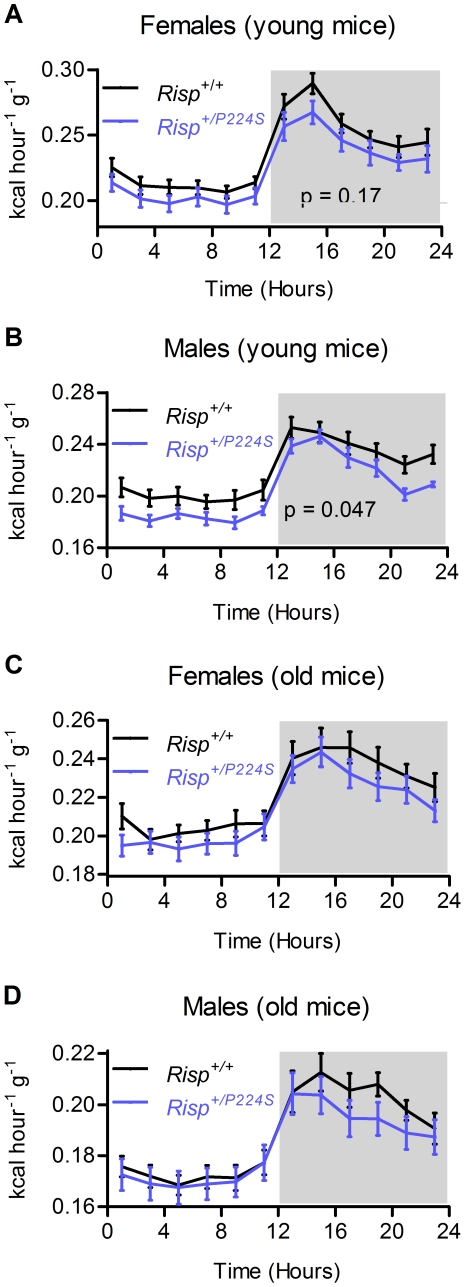
Metabolic rate as assessed by indirect calorimetry performed over a 24 hour period. The dark period is shaded. (**A**) Metabolic rate for 3-month-old female mice (n = 13). (**B**) Metabolic rate for 3-month-old male mice (n = 12). (**C**) Metabolic rate for 2-year-old female mice (n = 19 and 21). (**D**) Metabolic rate for 2-year-old male mice (n = 17 and 18).

The tissue- and substrate-specificity of the effect on mitochondrial respiration that we observed raised the question of whether or not the complex III defect of *Risp^+/P224S^* mice would have an physiological effect *in vivo*. We therefore sought to determine whether the overall metabolic rate of *Risp^+/P224S^* mutant mice was in fact affected by the mutation. Metabolic rate can be calculated from O_2_ consumption and CO_2_ production (i.e., indirect calorimetry). Indirect calorimetry offers a physiological measure of mitochondrial energy metabolism, since at least 90 percent of whole body oxygen consumption is mitochondrial in nature [28]. Metabolic rate over a 24-hour period was decreased by 7 percent in young *Risp^+/P224S^* male mice (p = 0.047); it was decreased by 5 percent in young females, although the difference did not reach statistical significance (Figure 4A and 4B). There was also a non-significant trend for a decrease in aged mutant mice, both males and females, for at least parts of the 24 hour cycle (Figure 4C and 4D). The respiratory exchange ratio (RER), the ratio between O_2_ consumption and CO_2_ production indicates whether carbohydrates or fatty acids are being used as metabolic fuels, The RER was not affected by *Risp* heterozygosity (results not shown). Thus, the impairment of complex III activity due to *Risp* heterozygosity can result in a physiologically significant impairment of mitochondrial function *in vivo* in young male mice, but we could not obtain strong statistical evidence for such an effect in females or aged mice.

### Biomarkers of oxidative stress are not affected by *Risp* heterozygosity

**Figure 5:**
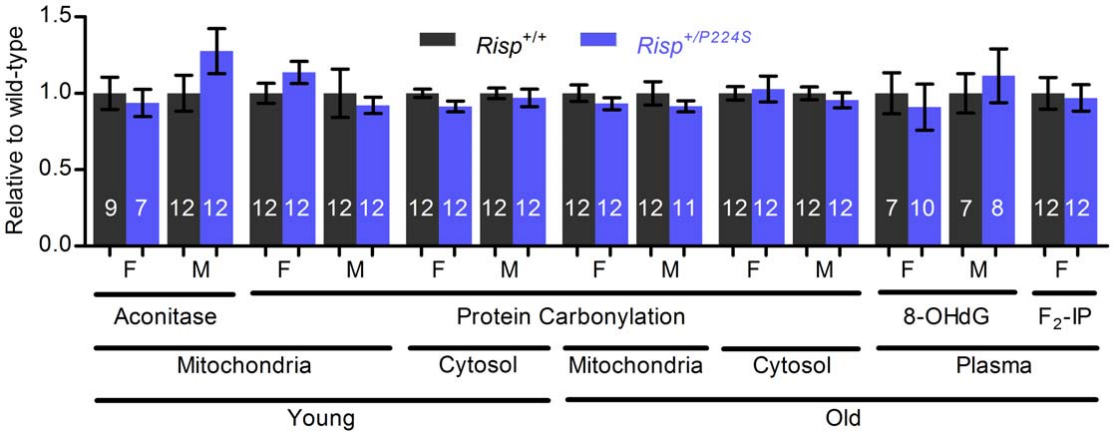
Biomarkers of oxidative stress are not affected by *Risp* heterozygosity. Various biomarkers of oxidative stress (enzymatic activity of aconitase, protein carbonylation, 8-OHdG and F_2_-isoprostanes) were measured in mitochondria, cytosol or plasma of young or old mice, as indicated. The sex of the mice is indicated below each bar (F for female, M for male). Values were normalized to a wild-type value of 1. Sample sizes are shown superimposed on each bar. Actual (not normalized) values are shown in Table S2.

Some form of accumulated damage is widely accepted as the causative factor behind aging. Oxidative stress, in particular reactive oxygen species (ROS) generated by the mitochondria, is often implicated (reviewed in [29]). Given the known tendency for inhibition of the ETC to increase mitochondrial ROS production [30], we wondered if *Risp* heterozygosity would induce oxidative stress. To answer this question, we measured several biomarkers of oxidative stress, in different cellular compartments and at different ages (Figure 5). Protein carbonylation (a product of oxidative modification to proteins) was not affected in the mitochondria or cytosol of *Risp^+/P224S^* mice of either sex at both young and old ages. Aconitase is an enzyme of the tricarboxylic acid cycle that is reversibly inactivated by ROS and is often used as a measure of *in vivo* oxidative stress [31]. Mitochondrial aconitase activity was not affected in young *Risp^+/P224S^* mice (it was not measured in old mice). Since it is not feasible to assay every tissue, we also determined the concentration of plasma F_2_-isoprostanes (a product of lipid peroxidation) and 8-OHdG (a product of DNA oxidation) as biomarkers of systemic oxidative stress [32], which have been previously shown to be affected in *Mclk1^+/−^* mice [17]. However, neither was found to be affected by *Risp* heterozygosity (F_2_-isoprostanes were only measured in female mice). Thus, it appears that the decreased complex III activity characterizing *Risp^+/P224S^* mice does not affect the level of oxidative stress in young or old mice.

### Effect of *Risp* heterozygosity on lifespan

**Figure 6:**
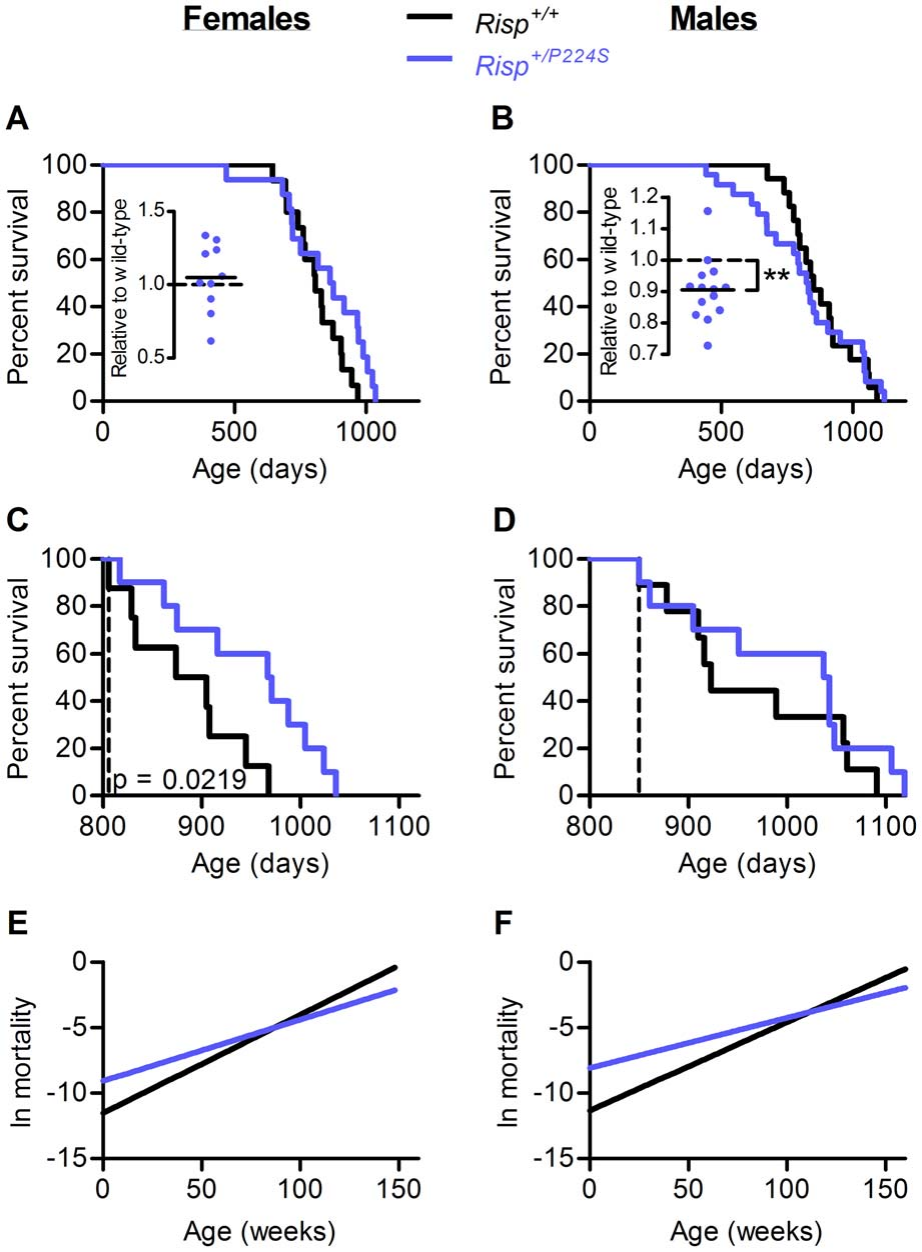
Lifespan and aging of *Risp* ^+/+^ and *Risp* ^+/P224S^ mice. (**A**) and (**B**) Effect of *Risp* heterozygosity on survival of female (15*^+/+^* and 16*^+/P224S^* mice) and male (17*^+/+^* and 24*^+/P224S^*) mice. Insets show the relative effect of *Risp* heterozygosity within litters. Each point therefore represents the average lifespan of all heterozygotes within a litter divided by the average lifespan of their wild-type littermates. By this measure, *Risp* heterozygosity shortened lifespan by 10 percent in males (p = 0.0037), with little effect in females. (**C**) and (**D**) show the remaining lifespan of *Risp^+/+^* and *Risp^+/P224S^* female and male mice that have survived to the median wild-type lifespan, indicated by the vertical dashed line (806 and 850 days for females and males, respectively). (**E**) and (**F**) Gompertz mortality curves for female and male mice. Both Gompertz parameters (the intrinsic vulnerability to death *A*, the y-intercept, and the age-dependent acceleration of mortality rate *k*, the slope) are statistically significantly different (p<0.05).

We allowed a group of male and female *Risp^+/P224S^* and ^+/+^ mice to live out their natural lifespan (Figure 6, Table S2). Wild-type males and females had median lifespans of 850 and 806 days respectively, comparable to what has been previously reported for mice on the C57BL/6 background [33]–[35]. Lifespans are often analyzed by comparing survival curves (as shown in Figure 6A and 6B). However, we took advantage of the fact that, because we study heterozygotes and use littermates as controls, all mice in the survival experiment had littermates of the opposite genotype. By comparing the average lifespans of wild-type and heterozygous littermates we determined the effect of *Risp* heterozygosity on lifespan within each litter, thereby accounting for inter-litter variability. By this measure, it was clear that *Risp^+/P224S^* males had an average lifespan 10 percent shorter than wild-type (Figure 6B, inset). In fact, in only 1 out of 13 litters did *Risp^+/P224S^* mice have a greater average lifespan than their wild-type littermates. There was no significant effect of *Risp* heterozygosity on average female lifespan within litters (Figure 6A, inset). Thus, lifespan is affected in a sex-specific manner in *Risp^+/P224S^* mice: average lifespan is shortened in males, and unaffected in females.

By visual inspection of the survival curves, *Risp* heterozygosity in females appeared to exert a beneficial effect on survival in the latter half of the lifespan. In fact, among mice that survive until the median wild-type lifespan, female *Risp^+/P224S^* mice will live 9% longer than wild-type (Figure 6C, p = 0.02 by log-rank test). Conversely, *Risp* heterozygosity appeared to exert its detrimental effect on survival of males in the earlier part of the survival curve (Figure 6D). Thus, the rate at which the risk of death increases with age appears to be decreased for both sexes in *Risp^+/P224S^* mice relative to wild-type. To test this, we fit the survival curves to the Gompertz function, which is commonly used to compare the mortality kinetics of different populations [36]. The Gompertz function yields two parameters, *A* and *k*, representing the intrinsic vulnerability to death (*A*) and the rate of acceleration of mortality with age (*k*). By this analysis, both female and male *Risp^+/P224S^* mice have an increased intrinsic vulnerability (represented by the y-intercept of Figure 6E and 6F), coupled with a decreased age-dependent mortality rate acceleration (represented by the slope) (both significant at p<0.05).

### Performance and pathology of aging mice

**Figure 7:**
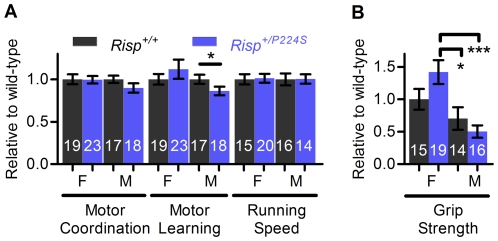
Performance of aged *Risp^+/P224S^* mice. The sex of the mice (F for female, M for male), as well as the type of test, are indicated below each bar. (**A**) Various measures of performance in aged, 2-year-old mice, including motor coordination (average of 3 trials after 2 days of training on a Rota-Rod), motor learning (Rota-Rod performance on day 3 relative to day 1), and running speed (the maximum speed mice could maintain on a powered treadmill). Because aged mice were not sacrificed in wild-type/heterozygous littermate pairs, values were normalized to a wild-type value of 1. (**B**) Grip strength (duration for which mice are capable of hanging suspended below a metal grid) for aged mice. Values were normalized to female wild-type. ‘*’ denotes p = 0.01 to 0.05 and ‘***’ denotes p<0.001 verses wild-type controls. Sample sizes are shown superimposed on each bar. Actual (non-normalized) values for (A) and (B) are shown in Table S2.

Aside from lifespan, the age-dependent decrease of health and physical performance is another important measure of aging [37]. To determine if *Risp^+/P224S^* mice age differently from wild-type we measured various indicators of performance and health in aged animals (2 years old). The maximum treadmill running speed (see Materials and Methods) of aged *Risp^+/P224S^* mice was not affected (Figure 7A). However, we found that aged *Risp^+/P224S^* males had impaired motor learning, coinciding with a trend for impaired motor performance (Figure 7A). Aged mice were also subjected to a simple test of grip strength and balance. This was done by measuring the time which they could hold themselves suspended beneath a wire grid. *Risp* heterozygosity appeared to increase grip strength in females and decrease it in males, but the differences did not reach statistical significance. However, *Risp* heterozygosity created a significant difference between males and females that was not present in wild-type mice, with a protective effect in females relative to males (Figure 7B). This is consistent with the relative effects of *Risp* heterozygosity on lifespan that we reported above.

Although mice were not subjected to a comprehensive necropsy, any evidence of gross pathology was noted at sacrifice. Several mice were found to have enlargements of one or more lymph nodes, symptomatic of lymphoma, the major age-related neoplastic pathology accounting for death in C57BL/6 mice [38]–[40]. Among apparently healthy mice sacrificed at 2 years of age, *Risp^+/P224S^* females appeared less likely to be affected by the condition than wild-type controls, although due to the relatively small number of animals affected this difference did not reach statistical significance (5/19 wild-type vs. 1/21 heterozygotes, p = 0.08 by Fisher's exact test). These mice all appeared outwardly healthy, with normal performance on the treadmill, Rota-Rod, and hanging-grid tests, indicating that the disease was at a relatively early stage. Since we did not conduct a complete analysis of pathology, it is possible that *Risp^+/P224S^* mice suffer from (or are protected from) other pathological conditions that went undetected in our study.

Body and organ weights were recorded at sacrifice of young and aged mice (Table S1). Brains from aged *Risp^+/P224S^* female mice weighed more than those from wild-type controls (0.49±0.004 vs. 0.47±0.008 g, p = 0.036); in contrast, brains from male *Risp^+/P224S^* mice weighed less than wild-type controls (0.47±0.007 vs. 0.48±0.004 g, p = 0.054). It is tempting to attribute this to a protective (female) or detrimental (male) effect of *Risp* heterozygosity against an age-related neurodegenerative process. For example, an age-dependent decrease of brain weight associated with neurodegeneration has been previously observed in a mouse model of accelerated aging [41]. Since brain weight was increased relative to that of young mice in all conditions, it is possible that this effect is due to differences in growth. However, there did not appear to be a generalized effect of *Risp* heterozygosity on growth: the weights of other organs were not affected in young or old mice, nor were overall body weights. Likewise, *Risp^+/P224S^* mice of both sexes reached the same maximum body weights at the same age as wild-type controls (Table S1). However, *Risp^+/P224S^* males exhibited a higher rate of aging-related weight loss (6.2±0.7 vs. 9.2±1 months for controls to lose 10 percent of peak weight, p = 0.016).

## Discussion

### A small loss of activity of complex III is sufficient to alter mitochondrial function

Several studies have used pharmaceutical inhibitors to show that inhibition of greater than 40–80 percent of complex III activity is required to substantially compromise respiratory function of mitochondria from various tissues [42]–[45]. This would imply that a complex III enzymatic deficiency of between 15 and 21 percent, as we observed in *Risp^+/P224S^* mice, should not affect mitochondrial function or produce in vivo effects. Although we found that mitochondrial oxygen consumption supported by glutamate + malate was not affected in mitochondria isolated from these mice, we found that succinate- and duroquinol-supported oxygen consumption of liver mitochondria was in fact decreased. In addition, this decrease in complex III activity was sufficient to significantly decrease the overall metabolic rate of young mutant males. Thus, it appears that complex III activity can be rate-limiting *in vivo*, and that pharmaceutical inhibition of the ETC *in vitro* may not completely mimic the effects of genetic variation on mitochondrial function.

It is unclear why succinate or duroquinol-supported respiration is particularly sensitive to a deficit in complex III. We hypothesize that this is related to the increased rate of O_2_ consumption (and therefore electron transfer) supported by these substrates, relative to glutamate + malate (apparent in Figure 3C). By this reasoning, the maximum rate of electron transfer supported by complex III from *Risp^+/P224S^* mice falls above that required for glutamate + malate supported respiration, but below that required for respiration supported by succinate or duroquinol. A similar effect has been reported previously, with succinate-supported mitochondrial respiration proving more sensitive to pharmaceutical inhibition of complex III than respiration supported by glutamate + malate [42].

Although *Risp* heterozygosity decreased complex III activity in heart mitochondria (Figure 2C), there did not appear to be any effect on mitochondrial oxygen consumption in this tissue, regardless of substrate used (Figure 3B). Interestingly, *Mclk1* ^+/−^ mice, with impaired electron transport between complexes I and III, show a similar phenotype: mitochondria from *Mclk1* ^+/−^ liver show decreased respiration, whereas mitochondria from heart are not affected [19].

### The response to ETC dysfunction is sex-dependent

Although the effects of *Risp* heterozygosity on enzymatic activity and mitochondrial oxygen consumption were comparable between sexes, metabolic rate was decreased to a greater extent in males than females. This coincided with a detrimental effect on male lifespan and with no effect on overall female lifespan. Sex-specific effects due to impairments of oxidative phosphorylation are not uncommon, with both male and female mice being reported as more severely affected by mitochondrial defects of similar magnitude [46]–[48]. Other, non-mitochondrial, interventions that affect lifespan have also been shown to be sex-specific. For example, impairment of the insulin/insulin-like signaling pathway in mice has been found to extend lifespan to a greater extent in females than males [49], [50].

It is striking that, although the effects on overall lifespan were different between the sexes, the effects on the Gompertz mortality rate parameters were similar: in both sexes, the presence of the P224S mutation increased vulnerability to death in the first part of the lifespan, while decreasing it in the later part. This effect was significant enough to result in an increased survival of *Risp^+/P224S^* females that survive to the median wild-type lifespan (Figure 6C). This, along with the fact that the five longest-lived female mice were all heterozygous (i.e., almost one-third of heterozygous mice lived longer than any of their wild-type controls), suggests an increased maximum lifespan of *Risp^+/P224S^* females. Furthermore, the log-rank p-value for the effect of *Risp* heterozygosity on female survival (Figure 6A) was 0.073, approaching statistical significance. It is therefore possible that future experiments with a greater sample size will reveal a more robust lifespan-extending effect of *Risp* heterozygosity in female mice.

The different effects on overall lifespan appear to be due to the greater increase in the intrinsic vulnerability to death of heterozygous males, relative to heterozygous females (Figure S1). It goes without saying that a major goal of future research must be to determine the reasons for these differences between the sexes.

### A sub-clinical impairment of ETC function can shorten mammalian lifespan

Severe mitochondrial dysfunction – mitochondrial disease – is known to have a negative impact on health, and can dramatically shorten lifespan [1], [2]. Although *Risp^+/P224S^* mice exhibit a measurable degree of mitochondrial dysfunction at a biochemical level, they don't exhibit any clinical signs of mitochondrial disease – for example, they perform as well as wild-type when forced to run at high speeds on a treadmill, and show no impairments to motor coordination or motor learning when young (Figure 1). A milder, age-dependent mitochondrial dysfunction has also been implicated in the aging process itself [6]. Our finding of a shortened lifespan in *Risp^+/P224S^* males with mild mitochondrial dysfunction, in the absence of apparent signs of mitochondrial disease, is compatible with that view. Metabolic rate, as an *in vivo* measure of mitochondrial function, was decreased most dramatically in young *Risp^+/P224S^* mice, which we found to be short-lived. In contrast, there were no statistically significant effects on metabolic rate in young *Risp^+/P224S^* female mice or in aged mice of either sex. Since *Risp* heterozygosity does not shorten lifespan in young females nor in aged females and males (Figure 6A, 6C and 6D), this is also consistent with a role for mitochondrial dysfunction in the shortened lifespan of *Risp^+/P224S^* male mice.

*Surf1^−/−^* [16] and *Mclk1^+/−^* mice [17], [18] do not appear to exhibit a lesser degree of mitochondrial dysfunction relative to *Risp^+/P224S^* mice, yet are long-lived. For example, *Mclk1^+/−^* mice exhibit decreased mitochondrial respiration with glutamate and malate in addition to succinate, and show increased levels of ROS damage [19]. *Mclk1* ^+/−^ mice also have a greater decrease in overall metabolic rate relative to *Risp^+/P224S^* mice (unpublished results). Although neuronal mitochondrial membrane potentials were unaltered in *Surf1^−/−^* mice, the effect on complex IV activity within heart and muscle tissue was within the range previously shown to impair mitochondrial function [43], and was substantially greater than the enzymatic impairments that characterize both *Risp^+/P224S^* and *Mclk1^+/−^* mice. Thus, the nature of the disruption of ETC activity may modulate the effects on lifespan. We have previously proposed that at least in some situations mitochondrial ROS acts as an intracellular messenger that activates stress-response mechanisms which can prolong lifespan in worms and in mice [23], [51]. The fact that *Risp^+/P224S^* mutants do not display increased ROS, at least as measured by markers of oxidative damage, is likely important in generating the lifespan effects that we observed.

Finally, in human mitochondrial diseases, the activity of one or more ETC complex is typically decreased to a much greater extent than the 14–22 percent we observed in *Risp^+/P224S^* mice. For example, individuals with an impairment of complex III due to mutations in the nuclear encoded gene BCS1L (involved in complex III assembly) were found to have lost 60–90 percent of complex III activity in affected tissues [52]. Liver complex III activity of affected individuals was found to be 53–64 nmoles mg^−1^ min^−1^, whereas that of apparently healthy control individuals ranged from 89 to 335 nmoles mg^−1^ min^−1^. Healthy individuals thus appeared to display a very wide range of levels of activities compatible with good health. *Risp^+/P224S^* mice also appeared outwardly healthy. However, the relatively minor decrease in complex III activity was sufficient to affect lifespan. This would suggest that, while people at the lower end of the control range may indeed have no clinical symptoms, their relatively low levels of ETC activity (analogous to the mild mitochondrial dysfunction of *Risp^+/P224S^* mice) could have important consequences for their long-term health and lifespan.

## Materials and Methods

### Creation of *Risp^+/P224S^* knock-in mice

Long-lived *isp-1(qm150)* worms contain a C to T transition, resulting in the conversion of proline 225 to serine [9]. “Knock-in” mice with the equivalent amino acid change (proline 224 to serine, in the second of the two *Risp* exons) were created by Ingenko (Australia). To do this, homologous recombination was used to replace the proline 224 codon CCC with TCG in C57BL/6 embryonic stem (ES) cells. A neomycin cassette flanked by Flp recombinase recognition target (FRT) sites located 0.7 kb 3′ of the final exon served as a selection marker. Selected ES cells were treated with Flp recombinase to excise the neomycin cassette; this excision was confirmed by PCR. These ES cells were microinjected to produce mice heterozygous for the *Risp* ^+^ and *Risp* ^P224S^ alleles. Sequencing of the translated region of exon 2 from *Risp^+/P224S^* mice confirmed the presence of the introduced point mutations, and that there was no further divergence from the wild-type and published sequences. However, sequencing of the remaining FRT site 3′ of the *Risp* gene revealed that 33 base-pairs of genomic DNA adjacent to the FRT sequence had been duplicated. This duplicated sequence flanked the FRT sequence.

### Animal husbandry

Mice were housed in a specific pathogen free facility at McGill University, 2–5 mice per cage. Mice in the lifespan experiment were bred by mating *Risp* ^+/−^ animals together. All other mice were produced by mating heterozygous males to wild-type females. To ensure genetic homogeneity, all breeders were descendents of the original group of *Risp^+/P224S^* mice. PCR amplification using primers flanking the remaining FRT site 3′ of the *Risp* gene was used for the routine identification of heterozygous mice. DNA from tails was used, with the following primers (5′ to 3′): CCAACTGATAAGACTATAGGC and GTCCATGACAGAGTCCTTCC. These produce a 333 base band from the knock-in allele and a 240 base band from the wild-type allele. Both bands are present in heterozygotes.

To determine the effects of heterozygosity for the P224S mutation on young mice, animals were sacrificed at 3–4 months of age. To determine the effect on aged mice, animals were sacrificed at 2 years of age. To determine how *Risp* heterozygosity affected lifespan, mice were kept until either natural death, or evidence of impending mortality necessitating euthanasia, as described previously [18]. Mice were euthanized by an overdose of isoflurane anesthetic. Blood was collected by cardiac puncture and plasma was prepared in EDTA-containing microtubes (Sarstedt), flash-frozen in liquid nitrogen and stored at −80°C in 100 µl aliquots. Tissues to be used for biochemical, antioxidant, or protein measurements were rapidly extracted, placed into cryotubes and flash-frozen in liquid nitrogen prior to storage at −80°C. Tissues to be used for measurements of mitochondrial respiration were kept on ice for a short period of time prior to homogenization and fractionation as described below. All studies were approved by the McGill Faculty of Science Animal Care Committee (protocol ID 4129) and conducted according to the guidelines of the Canadian Council on Animal Care.

### Quantification of RISP

Immunoblotting with a monoclonal anti-RISP antibody (MitoSciences # MS305) was used to quantify RISP in mitochondrial tissue fractions. An anti-porin antibody (Calbiochem # 529534) was used as a loading control. 8.5 µg of mitochondrial protein were loaded. Visualization of the bands was performed fluorescently using ECL Plus reagents and a Typhon Trio imager (GE Healthcare). Densitometry on the scanned image was performed with ImageJ (National Institutes of Health, USA).

### Isolation of Mitochondria

Tissues to be used for measurements of mitochondrial oxygen consumption were homogenized in 5 volumes (w/v) of homogenization buffer (0.3 M mannitol, 10 mM Tris, 1 mM EGTA, pH 7.4) for liver, or 3 ml of homogenization buffer (220 mM mannitol, 70 mM sucrose, 10 mM HEPES, 1 mM EGTA, 0.04 mM BSA, pH 7.4) for heart. Tissues to be used for biochemical assays or protein immunoblotting were homogenized in 0.25 M sucrose, 10 mM HEPES, 1 mM EDTA, pH 7.5. Homogenization buffer used for tissues intended for aconitase activity measurements was supplemented with 30 mM sodium citrate and 0.6 mM manganese chloride, and mitochondria were re-suspended in a solution containing 50 mM Tris-HCl, 1 mM cysteine, 1 mM citric acid and 0.5 mM MnCl_2_, pH 7.6. Mitochondria were isolated by differential centrifugation, as described previously [19], [53]. Protein concentrations were measured using the Bradford-based Biorad Protein Assay, using bovine serum albumin as a standard.

### Electron Transport Chain Complex Activity Assays

Mitochondria were disrupted by repeated freeze-thawing, and enzymatic assays were performed as previously described [54]. The one exception was the assay for complex IV activity, for which we observed a high baseline activity of the reaction mixture in the absence of mitochondria. To account for this, each measurement was accompanied by a blank containing only the reaction mixture. For each tissue-assay combination, preliminary experiments were conducted to determine concentrations within the linear range of detection. The final concentrations in each assay ranged from 1 to 10 µg/ml.

### Mitochondrial Oxygen Consumption

Oxygen consumption was measured polarographically with a Clark electrode connected to a recorder (Yellow Springs instruments) in a 1.75-ml water-jacketed closed chamber with magnetic stirring, at 30°C. Mitochondria were added to respiration buffer (125 mM sucrose, 65 mM KCl, 2 mM KH_2_PO_4_, 10 mM Hepes, pH 7.2) to a final concentration of 0.5 mg/ml for liver mitochondria and 0.15 mg/ml for heart mitochondria. 0.1% fatty acid-free BSA was added to the respiration buffer when duroquinol was being used as a substrate, and for succinate-supported respiration in liver mitochondria from aged mice.

Electron donor substrates were glutamate and malate (5 and 2.5 mM final concentrations, respectively) to support respiration through complex I, succinate (5 mM, with 2 µM rotenone) to support respiration through complex II, and duroquinol (approximately 0.5 mM) to support respiration through complex III [27]. Duroquinol was prepared by the addition of 2 M HCl and a mass of KBH_4_ to a duroquinone solution. Complete reduction was assumed when the yellow duroquinone solution became clear. ADP was added to a final concentration of 429 µM to induce state 3 respiration. For mitochondria from heart, 1.25 µg/ml oligomycin was added to end state 3 and initiate state 4. For mitochondria from liver, state 4 was initiated by ADP depletion.

The respiratory control ratio (RCR) was determined by dividing state 3 by state 4 respiration. By examining the initial oxygen consumption traces, it became apparent that some mitochondria from both genotypes exhibited abnormally poor respiratory control, presumably due to damage to the mitochondria during extraction. In order to eliminate these mitochondria from the analysis, we chose to exclude mitochondria for which the RCR for glutamate and malate stimulated respiration was less than 4, as this is the minimum RCR that is expected of intact, properly isolated mitochondria [55]. For mitochondria from aged mice, the RCR threshold was lowered to 3 in line with the natural decrease of RCR that has been observed with age [56]–[58].

### Biomarkers of Oxidative stress

Aconitase activity was measured as the reduction of NADP by isocitrate dehydrogenase as described [31]. Protein carbonylation was measured using a Protein Carbonyl Assay Kit (Cayman Chemical, catalog No. 10005020) as per the manufacturer's instructions. Plasma 8-hydroxy-2′-deoxyguanosine (8-OHdG) and F_2_-isoprostane levels were measured using enzyme immunoassay kits (from Assay Designs and Cayman Chemical, respectively). Plasma intended for measurement of isoprostanes was supplemented with 0.005% BHT before storage at −80°C to prevent spontaneous oxidative formation of F_2_-isoprostanes. An alkaline lysis procedure was performed on thawed plasma to liberate esterified F_2_-isoprostane, ensuring that both bound and un-bound F_2_-isoprostanes were measured in the assay.

### Indirect Calorimetry

Whole-body energy metabolism was measured with an indirect calorimetry system (Oxymax, Columbus Instruments). Mice were placed in the apparatus for 24 hours to allow them to acclimate before measurements were started. Oxygen consumption and carbon dioxide production was then recorded for 24 hours; these measurements were used to calculate energy usage [59]. We normalized metabolic rate to the combined weight of the liver, brain, heart and kidneys, because this method of normalization has been shown to best account for the rate of energy consumption [60]. Results were averaged over two hour intervals in order to smooth out the substantial point-to-point variation.

### Measures of performance and health

A Rota-Rod (UGO Basile) was used to measure motor coordination [61]. Mice were placed on the stationary rod which then accelerated from 2–80 rpm over a 5 minute period. The maximum speed at which mice were able to successfully maintain their position on the rotating rod was recorded. This protocol was performed three times per day over a three-day period, with mice given a 10 minute break in between runs. Grip strength was measured by timing how long a mouse was able to hold its body suspended below a wire grid [62], [63]. A motorized treadmill (Columbus Instruments) was used to measure exercise capacity. Mice were motivated to run by the presence of a metal grid at the rear of the treadmill which delivered a mild electric shock upon contact (approximately 1 mA, 5 times a second). Mice underwent a training protocol on day 1 of the experiment, and the experimental protocol on day 2. For young mice, the training protocol consisted of them being placed on the stationary treadmill for 10 minutes, then 10 minute intervals of 6, 10 and 14 m/min followed by another 10 minutes at 0 m/min. Aged mice (approximately 2 years) underwent essentially the same training protocol, but without the 14 m/min interval. The experimental protocol was used to determine the maximum speed at which mice were capable of running. Mice were placed on the stationary treadmill and allowed to acclimate for 10 minutes. The treadmill was then started at 6 m/min. Every minute thereafter the speed was increased by 2 m/min (young mice) or 1 m/min (old mice). The maximum speed which the mice could maintain for 1 minute was recorded. Mice were judged unable to continue if they touched the shock grid 15 times during a 1-minute interval or continuously for 5 seconds. Fertility of male mice was quantified by breeding *Risp^+/P224S^* or *^+/+^* males with wild-type females and recording the number of offspring born, up until the male breeders were 8 months of age.

### Statistics

Prism 5 (GraphPad Software) was used for all statistical analysis. Results are shown as average ± SEM. One-sample t-tests were used to determine whether the heterozygous/wild-type ratio was significantly different from 1. For the groups of mice sacrificed at 2 years of age, where it was not possible to consistently obtain wild-type and heterozygotes from the same litter, regular unpaired t-tests were use to test for differences between two groups. The non-parametric Mann Whitney test was used for motor learning due an obviously non-Gaussian distribution. One-way ANOVA was used to test for differences in grip strength among wild-type and heterozygous males and females. Repeated-measures two-way ANOVA was used to determine statistical significance for indirect calorimetry. Survival curves were compared using the log-rank (Mantel-Cox) test. Non-linear regression was used to determine the Gompertz parameters *A* (intrinsic vulnerability to death) and *k* (rate of acceleration of mortality with age). The following formula was used to fit survival data [64]:
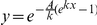
The linearized form of the Gompertz mortality rate function was used to graphically display the Gompertz parameters:
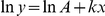



## Supporting Information

Table S1 Body and organ weights.
(XLS)

Table S2 Exact values for Figures 1, 2, 3, 5, 6 and 7.
(XLSX)

Table S3 State 4 and respiratory control ratios.
(XLS)

Figure S1 **Graphical depiction of the Gompertz parameters** ***A*** **and** ***k*** **.** (**A**) Intrinsic vulnerability to death (*A*) for females (F) and males (M). (**B**) Mortality rate acceleration (*k*) for females (F) and males (M). For both (A) and (B), the standard error is derived from the nonlinear fit of the Gompertz survival function.
(TIF)

## References

[1] DiMauro S, Schon EA (2003). Mitochondrial respiratory-chain diseases.. N Engl J Med.

[2] Edmond JC (2009). Mitochondrial disorders.. Int Ophthalmol Clin.

[3] Madamanchi NR, Runge MS (2007). Mitochondrial dysfunction in atherosclerosis.. Circ Res.

[4] Yap LP, Garcia JV, Han D, Cadenas E (2009). The energy-redox axis in aging and age-related neurodegeneration.. Advanced Drug Delivery Reviews.

[5] Lowell BB, Shulman GI (2005). Mitochondrial dysfunction and type 2 diabetes.. Science.

[6] Trifunovic A, Larsson NG (2008). Mitochondrial dysfunction as a cause of ageing.. Journal of Internal Medicine.

[7] Felkai S, Ewbank JJ, Lemieux J, Labbe JC, Brown GG (1999). CLK-1 controls respiration, behavior and aging in the nematode Caenorhabditis elegans.. Embo J.

[8] Ewbank JJ, Barnes TM, Lakowski B, Lussier M, Bussey H (1997). Structural and functional conservation of the Caenorhabditis elegans timing gene clk-1.. Science.

[9] Feng JL, Bussiere F, Hekimi S (2001). Mitochondrial electron transport is a key determinant of life span in Caenorhabditis elegans.. Developmental Cell.

[10] Van Raamsdonk JM, Hekimi S (2009). Deletion of the mitochondrial superoxide dismutase sod-2 extends lifespan in Caenorhabditis elegans.. PLoS Genet.

[11] Yang W, Hekimi S (2010). Two modes of mitochondrial dysfunction lead independently to lifespan extension in Caenorhabditis elegans.. Aging Cell.

[12] Dillin A, Hsu AL, Arantes-Oliveira N, Lehrer-Graiwer J, Hsin H (2002). Rates of behavior and aging specified by mitochondrial function during development.. Science.

[13] Lee SS, Lee RY, Fraser AG, Kamath RS, Ahringer J (2003). A systematic RNAi screen identifies a critical role for mitochondria in C. elegans longevity.. Nat Genet.

[14] Hamilton B, Dong Y, Shindo M, Liu W, Odell I (2005). A systematic RNAi screen for longevity genes in C. elegans.. Genes Dev.

[15] Copeland JM, Cho J, Lo T, Hur JH, Bahadorani S (2009). Extension of Drosophila Life Span by RNAi of the Mitochondrial Respiratory Chain.. Current Biology.

[16] Dell'agnello C, Leo S, Agostino A, Szabadkai G, Tiveron C (2007). Increased longevity and refractoriness to Ca(2+)-dependent neurodegeneration in Surf1 knockout mice.. Hum Mol Genet.

[17] Lapointe J, Stepanyan Z, Bigras E, Hekimi S (2009). Reversal of the mitochondrial phenotype and slow development of oxidative biomarkers of aging in long-lived Mclk1+/− mice.. J Biol Chem.

[18] Liu X, Jiang N, Hughes B, Bigras E, Shoubridge E (2005). Evolutionary conservation of the clk-1-dependent mechanism of longevity: loss of mclk1 increases cellular fitness and lifespan in mice.. Genes Dev.

[19] Lapointe J, Hekimi S (2008). Early mitochondrial dysfunction in long-lived Mclk1+/− mice.. J Biol Chem.

[20] Agostino A, Invernizzi F, Tiveron C, Fagiolari G, Prelle A (2003). Constitutive knockout of Surf1 is associated with high embryonic lethality, mitochondrial disease and cytochrome c oxidase deficiency in mice.. Human molecular genetics.

[21] Tiranti V, Hoertnagel K, Carrozzo R, Galimberti C, Munaro M (1998). Mutations of SURF-1 in Leigh Disease Associated with Cytochrome c Oxidase Deficiency.. The American Journal of Human Genetics.

[22] Turunen M, Olsson J, Dallner G (2004). Metabolism and function of coenzyme Q.. Biochim Biophys Acta.

[23] Wang DT, Malo D, Hekimi S (2010). Elevated Mitochondrial Reactive Oxygen Species Generation Affects the Immune Response via Hypoxia-Inducible Factor-1 alpha in Long-Lived Mclk1(+/−) Mouse Mutants.. Journal of Immunology.

[24] Zhang Z, Huang L, Shulmeister VM, Chi YI, Kim KK (1998). Electron transfer by domain movement in cytochrome bc1.. Nature.

[25] Iwata S, Lee JW, Okada K, Lee JK, Iwata M (1998). Complete structure of the 11-subunit bovine mitochondrial cytochrome bc1 complex.. Science.

[26] Gatti DL, Meinhardt SW, Ohnishi T, Tzagoloff A (1989). Structure and function of the mitochondrial bc1 complex. A mutational analysis of the yeast Rieske iron-sulfur protein.. J Mol Biol.

[27] Kayser E-B, Sedensky MM, Morgan PG, Hoppel CL (2004). Mitochondrial Oxidative Phosphorylation Is Defective in the Long-lived Mutant clk-1.. J Biol Chem.

[28] Rolfe DF, Brown GC (1997). Cellular energy utilization and molecular origin of standard metabolic rate in mammals.. Physiol Rev.

[29] Balaban RS, Nemoto S, Finkel T (2005). Mitochondria, oxidants, and aging.. Cell.

[30] Turrens JF (1997). Superoxide Production by the Mitochondrial Respiratory Chain.. Bioscience Reports.

[31] Hausladen A, Fridovich I (1996). Measuring nitric oxide and superoxide: rate constants for aconitase reactivity.. Methods Enzymol.

[32] Arguelles S, Garcia S, Maldonado M, Machado A, Ayala A (2004). Do the serum oxidative stress biomarkers provide a reasonable index of the general oxidative stress status?. Biochim Biophys Acta.

[33] Gates AC, Bernal-Mizrachi C, Chinault SL, Feng C, Schneider JG (2007). Respiratory uncoupling in skeletal muscle delays death and diminishes age-related disease.. Cell Metab.

[34] Cai WJ, He JC, Zhu L, Chen X, Wallenstein S (2007). Reduced oxidant stress and extended lifespan in mice exposed to a low glycotoxin diet - Association with increased AGER1 expression.. American Journal of Pathology.

[35] Yuan R, Tsaih SW, Petkova SB, de Evsikova CM, Xing SQ (2009). Aging in inbred strains of mice: study design and interim report on median lifespans and circulating IGF1 levels.. Aging Cell.

[36] Finch CE (1990). Longevity, Senescence, and the Genome.

[37] Thouvarecq R, Protais P, Jouen F, Caston J (2001). Influence of cholinergic system on motor learning during aging in mice.. Behav Brain Res.

[38] Ran Q, Liang HY, Ikeno Y, Qi WB, Prolla TA (2007). Reduction in glutathione peroxidase 4 increases life span through increased sensitivity to apoptosis.. Journals of Gerontology Series a-Biological Sciences and Medical Sciences.

[39] Jang YC, Perez VI, Song W, Lustgarten MS, Salmon AB (2009). Overexpression of Mn superoxide dismutase does not increase life span in mice.. J Gerontol A Biol Sci Med Sci.

[40] Zhang Y, Ikeno Y, Qi W, Chaudhuri A, Li Y (2009). Mice deficient in both Mn superoxide dismutase and glutathione peroxidase-1 have increased oxidative damage and a greater incidence of pathology but no reduction in longevity.. J Gerontol A Biol Sci Med Sci.

[41] Shimada A, Ohta A, Akiguchi I, Takeda T (1992). Inbred SAM-P/10 as a mouse model of spontaneous, inherited brain atrophy.. Journal of neuropathology and experimental neurology.

[42] Taylor RW, Birch-Machin MA, Bartlett K, Lowerson SA, Turnbull DM (1994). The control of mitochondrial oxidations by complex III in rat muscle and liver mitochondria. Implications for our understanding of mitochondrial cytopathies in man.. J Biol Chem.

[43] Rossignol R, Malgat M, Mazat JP, Letellier T (1999). Threshold effect and tissue specificity. Implication for mitochondrial cytopathies.. J Biol Chem.

[44] Davey GP, Clark JB (1996). Threshold Effects and Control of Oxidative Phosphorylation in Nonsynaptic Rat Brain Mitochondria.. Journal of Neurochemistry.

[45] Davey GP, Peuchen S, Clark JB (1998). Energy thresholds in brain mitochondria. Potential involvement in neurodegeneration.. J Biol Chem.

[46] Diaz F, Thomas CK, Garcia S, Hernandez D, Moraes CT (2005). Mice lacking COX10 in skeletal muscle recapitulate the phenotype of progressive mitochondrial myopathies associated with cytochrome c oxidase deficiency.. Hum Mol Genet.

[47] Graham BH, Waymire KG, Cottrell B, Trounce IA, MacGregor GR (1997). A mouse model for mitochondrial myopathy and cardiomyopathy resulting from a deficiency in the heart/muscle isoform of the adenine nucleotide translocator.. Nat Genet.

[48] Yang H, Brosel S, Acin-Perez R, Slavkovich V, Nishino I (2010). Analysis of mouse models of cytochrome c oxidase deficiency owing to mutations in Sco2.. Hum Mol Genet.

[49] Holzenberger M, Dupont J, Ducos B, Leneuve P, Geloen A (2003). IGF-1 receptor regulates lifespan and resistance to oxidative stress in mice.. Nature.

[50] Selman C, Lingard S, Choudhury AI, Batterham RL, Claret M (2008). Evidence for lifespan extension and delayed age-related biomarkers in insulin receptor substrate 1 null mice.. Faseb Journal.

[51] Yang W, Hekimi S (2010). A mitochondrial superoxide signal triggers increased longevity in Caenorhabditis elegans.. PLoS Biol.

[52] de Lonlay P, Valnot I, Barrientos A, Gorbatyuk M, Tzagoloff A (2001). A mutant mitochondrial respiratory chain assembly protein causes complex III deficiency in patients with tubulopathy, encephalopathy and liver failure.. Nat Genet.

[53] Zini R, Berdeaux A, Morin D (2007). The differential effects of superoxide anion, hydrogen peroxide and hydroxyl radical on cardiac mitochondrial oxidative phosphorylation.. Free Radic Res.

[54] Kwong LK, Sohal RS (2000). Age-related changes in activities of mitochondrial electron transport complexes in various tissues of the mouse.. Arch Biochem Biophys.

[55] Pallotti F, Lenaz G (2001). Isolation and subfractionation of mitochondria from animal cells and tissue culture lines.. Methods Cell Biol.

[56] Ventura B, Genova ML, Bovina C, Formiggini G, Lenaz G (2002). Control of oxidative phosphorylation by Complex I in rat liver mitochondria: implications for aging.. Biochim Biophys Acta.

[57] Hagen TM, Yowe DL, Bartholomew JC, Wehr CM, Do KL (1997). Mitochondrial decay in hepatocytes from old rats: membrane potential declines, heterogeneity and oxidants increase.. Proc Natl Acad Sci U S A.

[58] Horton AA, Spencer JA (1981). Decline in respiratory control ratio of rat liver mitochondria in old age.. Mech Ageing Dev.

[59] McLean JA, Tobin G (1987). Animal and Human Calorimetry.

[60] Greenberg JA, Boozer CN (2000). Metabolic mass, metabolic rate, caloric restriction, and aging in male Fischer 344 rats.. Mech Ageing Dev.

[61] Buitrago MM, Schulz JB, Dichgans J, Luft AR (2004). Short and long-term motor skill learning in an accelerated rotarod training paradigm.. Neurobiol Learn Mem.

[62] Crawley JN (2000). Motor Functions. What's wrong with my mouse? : behavioral phenotyping of transgenic and knockout mice.

[63] Sango K, McDonald MP, Crawley JN, Mack ML, Tifft CJ (1996). Mice lacking both subunits of lysosomal beta-hexosaminidase display gangliosidosis and mucopolysaccharidosis.. Nat Genet.

[64] Doubal S, Klemera P (1997). Practical methodology of evaluation of mortality curves and detection of aging-related interventions.. AGE.

