# Supplementary figures and images for: A Mild Impairment of Mitochondrial Electron Transport Has Sex-Specific Effects on Lifespan and Aging in Mice
Source: PLoS One. 2011 Oct 10;6(10):e26116. doi: 10.1371/journal.pone.0026116 (PMC3189954; doi:10.1371/journal.pone.0026116)

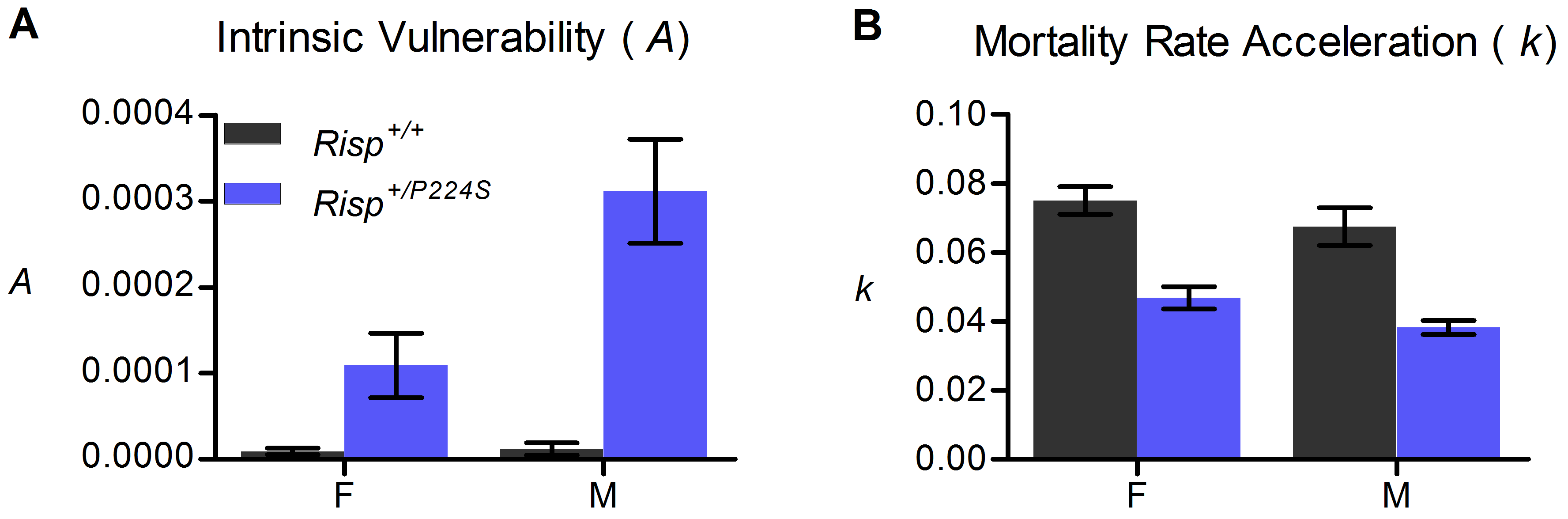

Supplement: Figure S1 — Graphical depiction of the Gompertz parameters A and k . (A) Intrinsic vulnerability to death (A) for females (F) and males (M). (B) Mortality rate acceleration (k) for females (F) and males (M). For both (A) and (B), the standard error is derived from the nonlinear fit of the Gompertz survival function. (TIF) [file pone.0026116.s004.tif]
